# Implantable Cardiac Defibrillator Lead Infective Endocarditis Due to *Rothia* Specie: A Rare Case in An Immunocompetent Man

**DOI:** 10.31083/j.rcm2305149

**Published:** 2022-04-24

**Authors:** Chukwuemeka A. Obi, Obiora Egbuche, Shirley I. Nwokike, Kenechukwu Mezue, Temidayo Abe, Kishen Bulsara, Titilope Olanipekun, Ifeoma Onuorah

**Affiliations:** ^1^Division of Cardiovascular Medicine, Medical University of South Carolina, Charleston, SC 29425, USA; ^2^Division of Cardiovascular Medicine, Ohio State University, Columbus, OH 43210, USA; ^3^Department of Internal Medicine, Medical College of Georgia, Augusta, GA 30912, USA; ^4^Division of Nuclear Cardiology, Massachusetts General Hospital, Harvard Medical School, Boston, MA 02114, USA; ^5^Department of Internal Medicine, Morehouse School of Medicine, Atlanta, GA 30310, USA; ^6^Department of Internal Medicine, Donald and Barbara Zucker School of Medicine at Hofstra Northwel, Hampstead, NY 11549, USA; ^7^Department of Hospital Medicine, Covenant Health System, Knoxville, TN 37922, USA; ^8^Division of Cardiovascular Disease, Emory University School of Medicine, Atlanta, GA 30322, USA

**Keywords:** Cardiac defibrillator lead infection, Infective endocarditis, *Rothia dentocariosa*, Imunocompetent host

## Abstract

**Background:**

*Rothia* species are known to cause dental caries and periodontal disease, and infrequently cause native or prosthetic valve endocarditis mostly in immunocompromised persons. With an increasing use of implantable cardiac devices, early clinical suspicion and a rapid diagnosis of endocarditis is essential for optimal treatment to reduce complications and mortality. Bacteremic infection with *Rothia dentocariosa* in immunocompetent persons is uncommon. Pacemaker lead-related endocarditis caused by Rothia spp. is rare and management guidelines are not defined

**Case Presentation:**

We report a rare case of implantable cardiac defibrillator (ICD) lead endocarditis in an immunocompetent patient that was caused by *Rothia dentocariosa*.

**Conclusions:**

Clinicians should be aware of this rare cause of CIED lead infections and should be acquainted with the optimal strategies of prompt antibiotic therapy and removal of the infected device/leads.

## 1. Introduction

Cardiovascular implantable electronic devices (CIED) related infective endocarditis is a serious complication of implantable cardiac device and is associated with a high rate of mortality and morbidities. The most common pathogens is *Staphylococcus aureus* causing acute endocarditis, and *Streptococcus viridans *causing subacute endocarditis. With an increasing use of implantable cardiac devices, early clinical suspicion and a rapid diagnosis of endocarditis is essential for optimal treatment to reduce complications and mortality. *Rothia dentocariosa *is a gram-positive rod commonly found as part of the normal flora of the human oropharynx and upper respiratory tract that can cause a wide range of diseases in immunocompromised patients. However it rarely causes disease in immunocompetent patients. Pacemaker infection with *Rothia* species is infrequently reported. We present a rare case of implantable cardiac defibrillator (ICD) lead infective endocarditis where the causative pathogen isolated was* Rothia dentocariosa.*

## 2. Clinical Presentation

**Fig. 1.**
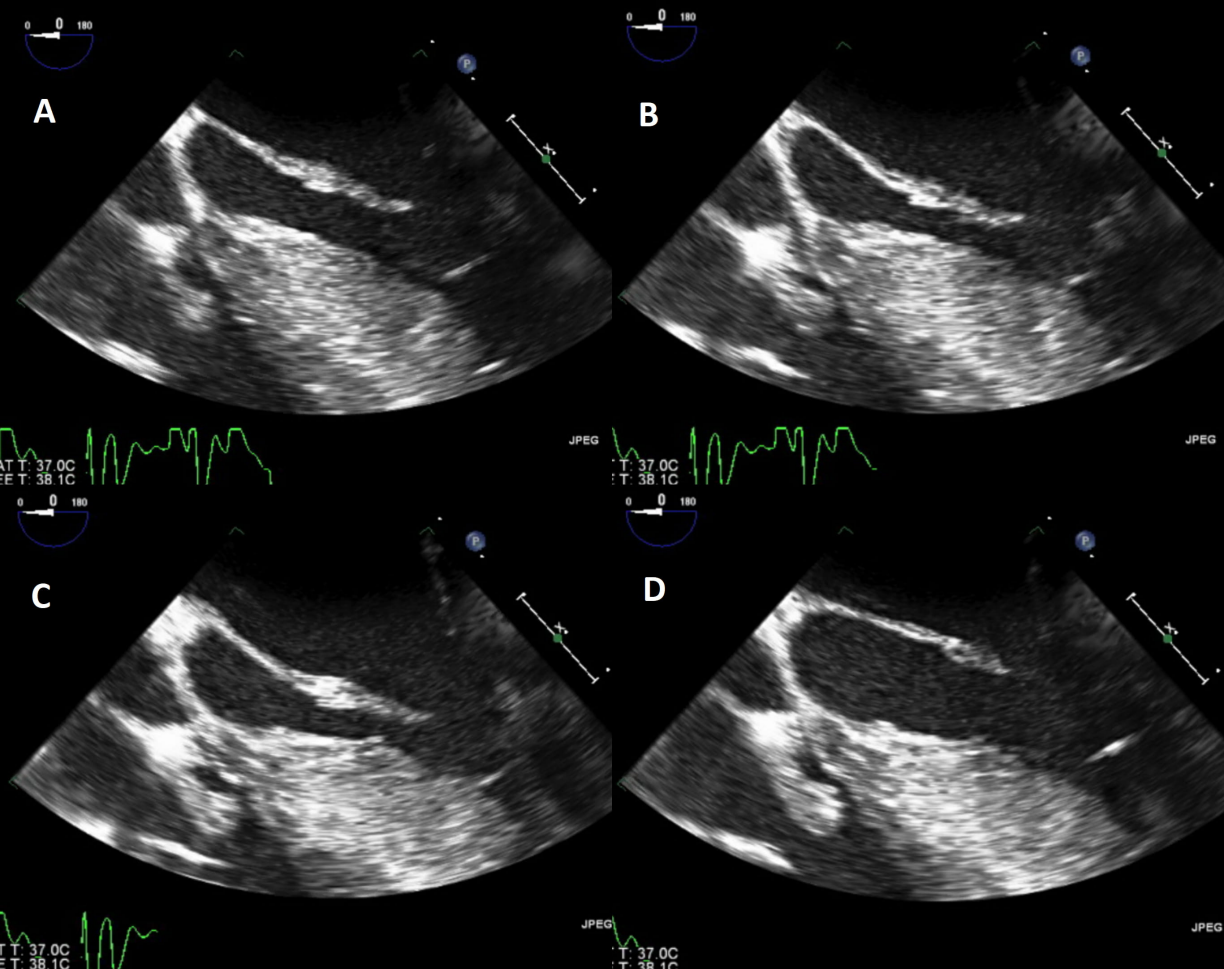
**(A–D) Transesophageal echocardiogram at mid-esophagus showing different still-frame images of vegetation attached to device lead just above the level of the tricuspid valve**. (A) Still-frame image of vegetation on tricuspid valve. (B) Still-frame image of vegetation on tricuspid valve at zero degree. (C) TEE image of vegetation on tricuspid valve. At 29 degree (D) TEE image of vegetation at 75 degree.

**Fig. 2.**
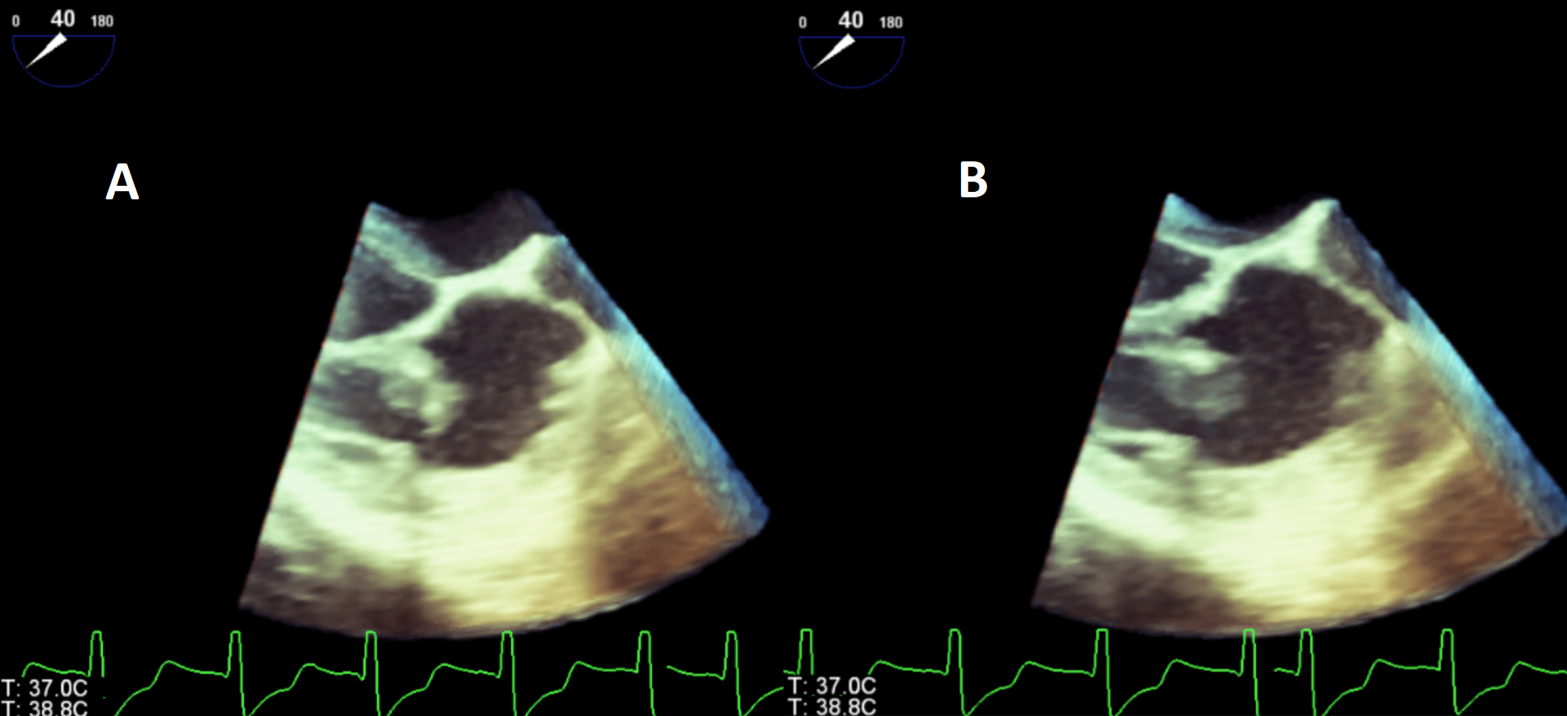
**(A–B) Transesophageal echocardiogram at mid-esophagus showing different still-frame 3D-images of vegetation prolapsing in and out of the right ventricle**. (A) Vegetation prolapsing in and out the right ventricle. (B) Tricuspid valve vegetation prolapsing in and out the right ventricle.

We present a 33-year-old male with hypertrophic obstructive cardiomyopathy (HOCM) who was admitted for fever and chest pain for 2 weeks. Five years prior to presentation, an implantable cardioverter defibrillator (ICD) was implanted for unexplained syncope in the setting of HOCM. Patient had initially presented to another hospital emergency department with two weeks of fever, rigors and chills. Blood cultures were not drawn, and no antibiotic was administered. He was treated conservatively for a viral syndrome and discharged. He then presented to our emergency department, this time with malaise, chest discomfort, and high-grade fever. Physical examination was remarkable for a 2/6 systolic murmur heard diffusely over the precordium. Initial temperature was noted to be 102 degrees Fahrenheit and a pulse rate of 104 beats per minute. His initial laboratory results revealed total leukocyte count of 15,600 with 82.3% neutrophils. Troponin I was 0.13 ng/mL and electrocardiogram showed sinus tachycardia and left ventricular hypertrophy. Blood culture grew *Rothia dentocariosa* in 2 separate bottles which was sensitive to penicillin and vancomycin. Transthoracic echocardiography was significant for a hyperdynamic left ventricle, asymmetric septal hypertrophy, mild mitral regurgitation, systolic anterior motion of the mitral valve leaflet with a left ventricular outflow tract resting gradient of 90 mmHg but no vegetation was noted. There was mild tricuspid regurgitation and the pulmonic valve was normal. Given that he had an ICD and persistent bacteremia for 2 weeks, a transesophageal echocardiography (TEE) was obtained and showed a 1.8 × 1.0 cm mobile vegetation localized to the ICD lead just above the level of the tricuspid valve (Fig. 1) and (Fig. 2). There was no native valve involvement. A clinical diagnosis of definite infective endocarditis was made using the modified Duke criteria.

The patient was started on vancomycin (Pfizer, Rocky Mount, NC, USA), gentamicin (Pfizer, Rocky Mount, NC, USA), and rifampin (Sanofi Pharmaceuticals., Bridgewater Township, NJ, USA). He was then transferred to another hospital for percutaneous ICD lead extraction with laser. He underwent successful explantation of the ICD lead, and repeat blood cultures were negative. He completed a 6-week course of antibiotics, including a 2-week course of parenteral antibiotics after device was extracted. Prior to being discharged from the hospital a subcutaneous ICD was implanted. His post-discharge course was uneventful and he now follows at our cardiac outpatient clinic.

## 3. Discussion

*Rothia dentocariosa* is a gram-positive rod found commonly as part of the normal flora of the mouth. *Rothia *species are commonly associated with dental caries and periodontal disease. It can cause invasive diseases like meningitis and endocarditis in immunocompromised patients, but rarely reported in immunocompetent patients. The incidence of cardiac device infection ranges from 0.13% to 19.9% [1]. According to a publication by the American Heart Association (AHA), staphylococcus species cause 60%–80% of cardiovascular implantable electronic device infections (CIED) [2]. As a result, vancomycin should be empirically administered until microbiological results are known.

Although a few cases of *Rothia dentocariosa* endocarditis have been reported [3], cardiovascular implantable electronic device lead infection with *Rothia* has not been previously reported thus its clinical features, optimal management and prognosis remain unknown. Our patient was immunocompetent without recent dental procedure or periodontal disease, but had *Rothia* species ICD lead endocarditis complicated by myopericarditis that was successfully treated with antibiotic therapy and a delayed surgical explantation. He had complete resolution of infection without any evidence of systemic embolization despite a large vegetation and delayed surgical intervention.

According to the AHA, early removal of CIED and antibiotics therapy is recommended in patients with established CIED infection [2]. This is because infection relapse can be as high as 7.3% in patients with retained indwelling device [4]. However, a systematic review of published cases of Rothia infective endocarditis suggest a favorable prognosis [5]. Up to 2 weeks of parenteral antibiotics therapy after hardware removal is recommended [2]. In patients with CIED infection who are not candidates for or who decline device explantation, long-term suppressive therapy should be considered [2]. Extended duration for long-term suppressive antibiotic therapy may become warranted if the infective process persists. Data regarding clinical outcomes of long-term suppressive therapy for CIED infections is limited.

## 4. Conclusions

In this case report, we acknowledge a rare case of ICD lead endocarditis caused by *Rothia dentocariosa* in an immunocompetent host. Clinicians should be aware of this rare cause of CIED lead infections and should be acquainted with the optimal strategies of prompt antibiotic therapy and removal of the infected device/leads. The use of long-term suppressive antibiotic as a therapeutic strategy is an option for patients who decline device explantation. Future studies on the optimal choice and dosing of antibiotics to prevent relapse in patients with CIED lead endocarditis who decline or are not candidates for complete device explantation are needed.
